# CD137^+^ and regulatory T cells as independent prognostic factors of survival in advanced non-oncogene addicted NSCLC patients treated with immunotherapy as first-line

**DOI:** 10.1186/s12967-024-05142-6

**Published:** 2024-04-03

**Authors:** Alain Gelibter, Angela Asquino, Lidia Strigari, Ilaria Grazia Zizzari, Lucrezia Tuosto, Fabio Scirocchi, Angelica Pace, Marco Siringo, Elisa Tramontano, Serena Bianchini, Filippo Bellati, Andrea Botticelli, Donatella Paoli, Daniele Santini, Marianna Nuti, Aurelia Rughetti, Chiara Napoletano

**Affiliations:** 1 Division of Oncology, Department of Radiological, Oncological and Pathological Science, Policlinico Umberto I, “Sapienza” University of Rome, Rome, Italy; 2 Laboratory of Tumor Immunology and Cell Therapies, Department of Experimental Medicine, Sapienza University of Rome, Viale Regina Elena 324, 00161 Rome, Italy; 3 Department of Medical Physics, IRCCS Azienda Ospedaliera-Universitaria Di Bologna, 40138 Bologna, Italy; 4 Laboratory of Seminology-Sperm Bank “Loredana Gandini”, Department of Experimental Medicine, “Sapienza” University of Rome, 00161 Rome, Italy; 5 Department of Medical and Surgical Sciences and Translational Medicine, Sant’Andrea University Hospital, Sapienza University of Rome, Via Di Grottarossa 1035, 00189 Rome, Italy

**Keywords:** NSCLC, Immune checkpoint inhibitors, Pembrolizumab, Immunotherapy, CD137^+^ T cells, Tregs, MDSCs, Soluble immune checkpoints

## Abstract

**Background:**

Immune checkpoint inhibitors (ICIs), administered alone or combined with chemotherapy, are the standard of care in advanced non-oncogene addicted Non-Small Cell Lung Cancer (NSCLC). Despite these treatments' success, most long-term survival benefit is restricted to approximately 20% of patients, highlighting the need to identify novel biomarkers to optimize treatment strategies. In several solid tumors, immune soluble factors, the activatory CD137^+^ Tcells, and the immunosuppressive cell subsets Tregs and MDSCs (PMN(Lox1^+^)-MDSC and M-MDSCs) correlated with responses to ICIs and clinical outcomes thus becoming appealing predictive and prognostic factors. This study investigated the role of distinct CD137^+^ Tcell subsets, Tregs, MDSCs, and immune-soluble factors in NSCLC patients as possible biomarkers.

**Methods:**

The levels of T cells, MDSCs and soluble factors were evaluated in 89 metastatic NSCLC patients who underwent ICIs as first- or second-line treatment. T cell analysis was performed by cytoflurimetry evaluating Tregs and different CD137^+^ Tcell subsets also combined with CD3^+^, CD8^+^, PD1^+^, and Ki67^+^ markers. Circulating cytokines and immune checkpoints were also evaluated by Luminex analysis. All these parameters were correlated with several clinical factors (age, sex, smoking status, PS and TPS), response to therapy, PFS , and OS . The analyses were conducted in the overall population and in patients treated with ICIs as first-line (naïve patients).

**Results:**

In both groups of patients, high levels of circulating CD137^+^ and CD137^+^PD1^+^ T cells (total, CD4 and CD8) and the soluble factor LAG3 positively correlated with response to therapy. In naïve patients, PMN(Lox1^+^)-MDSCs negatively correlated with clinical response, and a high percentage of Tregs was associated with favorable survival. Moreover, the balance between Treg/CD137^+^ Tcells or PMN(Lox1^+^)-MDSC/CD137^+^ Tcells was higher in non-responding patients and was associated with poor survival. CD137^+^ Tcells and Tregs resulted as two positive independent prognostic factors.

**Conclusion:**

High levels of CD137^+^, CD137^+^PD1^+^ Tcells and sLAG3 could predict the response to ICIs in NSCLC patients independently by previous therapy. Combining the evaluation of CD137^+^ Tcells and Tregs also as Treg/CD137^+^ T cells ratio it is possible to identify naive patients with longer survival.

**Supplementary Information:**

The online version contains supplementary material available at 10.1186/s12967-024-05142-6.

## Background

Non-small cell lung cancer (NSCLC) represents 85% of newly diagnosed lung cancers. Most of these patients are initially diagnosed with advanced disease and receive different treatments according to the stage of the disease and the presence of oncogenic driver mutations [1]. The introduction of immune checkpoint inhibitors (ICIs) has changed the clinical outcome of these patients [2]. In the advanced non-oncogene addicted NSCLC group, the anti-PD1/PD-L1 inhibitors (Nivolumab, Pembrolizumab, Atezolizumab) were firstly approved as second-line treatment after progression to platinum-doublet chemotherapy [3–5]. In the last years, PD1 axis blockade with pembrolizumab represents the standard of care in this setting of patients as monotherapy or combination according to the tumor PDL-1 expression (defined as Tumor Proportion Score (TPS)). Indeed, pembrolizumab alone is administered in patients with TPS ≥ 50% [6], while patients with TPS < 50% are treated with platinum doublets with pemetrexed or paclitaxel plus ICIs (pembrolizumab or nivolumab plus ipilimumab, an anti-CTLA4 antibody) [7, 8]. Therefore, the tumor expression of PDL-1 (tPDL-1) is currently the only biomarker to guide the therapeutic choice, but with low efficiency, highlighting the need to identify new biomarkers to improve the efficacy of the well-consolidated tPDL1.

Although the critical role of the PD1/PDL-1 axis in the initial phase of tumor progression, several other immune parameters were evaluated in NSCLC patients as possible biomarkers not only in the tumor, but also in the peripheral blood. Beyond neutrophils- or myeloid- or platelet-to-lymphocytes ratio correlated with survival to ICIs, other circulating immune cells were considered [9–11]. NSCLC patients responding to anti-PD1 therapy showed increasing levels of circulating CD8^+^PD1^+^ T cells with an effector phenotype [12]. The ratio of regulatory T cells (Tregs) to Lox1^+^-PMN-MDSCs predicted the response in Nivolumab-treated NSCLC patients [13]. We recently demonstrated the predictive and prognostic role of CD137^+^ T cell subset in solid tumors, including NSCLC, under anti-PD1 treatment [14, 15]. This population was recognized as naturally occurring antigen-specific T cells and was associated with the wellness of the immune system [14, 16].

Several other studies also evaluated the role of soluble immune modulators, such as checkpoints and cytokines, as possible biomarkers for NSCLC patients. Among the immune checkpoints, the soluble PD-1 and PDL-1 were the most investigated, however, their role as predictive and prognostic factors was controversial [17]. Our previous analysis showed that soluble PDL-1 was a negative prognostic factor for immunotherapy [18], especially in NSCLC patients. Moreover, we demonstrated that in NSCLC patients treated with nivolumab, the soluble PD1 decreased after therapy in responder patients, while non-responders showed increasing levels of the soluble LAG3 [19]. Among cytokines, IL8 and IL6 seemed to have an important role in tumor growth modulating the response to treatments. Indeed, several studies demonstrated that the pro-inflammatory cytokine IL8 increased upon progression in NSCLC patients [20] and that together with IL6 predicted the response to immunotherapy [21].

All these data underlined the critical role of immune cells and soluble factors in the response to ICI, making both these components optimal candidates to identify those patients who will benefit from therapy.

In this study, we analyzed before the beginning of immunotherapy, the levels of different circulating T cell subsets and soluble immune modulators (checkpoints and cytokines) of 89 advanced NSCLC patients under ICI treatment as first- and second-line therapy to identify new predictive and prognostic biomarkers. The analyses of the immune cells were focused particularly on evaluating different CD137^+^ T cell subsets as populations representing the activation state of the immune system. On the contrary, Tregs and MDSCs (both Lox1^+^PMN-MDSCs and Monocyte-MDSCs (M-MDSCs) were employed as indicators of immune suppression. These parameters (cellular and soluble) were combined with clinical characteristics (age, sex, performance status (PS), TPS, and smoking status) and correlated with response to therapy, progression-free survival (PFS), and overall survival (OS).

## Methods

### Patients’ characteristics

Between 2016 and 2023, this study enrolled patients with a confirmed diagnosis of metastatic NSCLC who underwent treatment with anti-PD1 with or without chemotherapy as first or second-line treatment at the Medical Oncology Department of Policlinico Umberto I Hospital. Patients were treated with ICI monotherapy with pembrolizumab (as first-line in patients with TPS ≥ 50) or nivolumab (as second-line unregard less TPS status) with standard dose and schedule until disease progression or unacceptable toxicity. Alternatively, patients with TPS < 50% were treated either with Pembrolizumab and chemotherapy or Nivolumab plus Ipilimumab (an anti-CTLA4 antibody) associated with a short course of chemotherapy.

Criteria of inclusion were: age > 18 years; histologically documented diagnosis of NSCLC; adequate cardiac, pulmonary, renal, liver, and bone marrow function; Eastern Cooperative Oncology Group (ECOG) Performance Status (PS) scored between 0 and 2; patients with symptomatic and stable central nervous system metastases. Criteria of exclusion were: symptomatic interstitial lung disease and any other significant comorbidity, autoimmune disease, and systemic immunosuppression. The response was assessed every 3 months until documented disease progression using immune-related Response. Evaluation Criteria in Solid Tumors (i-RECIST) PFS, OS, and Disease Control Rate (DCR) were evaluated. PFS was defined as the time from the beginning of anti-PD1 therapy until tumor progression or death from any cause. OS was defined as the interval between the immunotherapy starts to death for any case. The DCR was used to evaluate the response to ICI. Responders (R) identified patients with a complete or partial response or stable disease after 3 months of treatment, while non-responders (NR) represented patients who progressed within 3 months. The study was conducted following good clinical practice guidelines and the declaration of Helsinki and was approved by the Ethics Committee of Policlinico Umberto I (Ethical Committee Protocol, RIF.CE: 4181).

### Peripheral blood mononuclear cell and serum collection

Peripheral blood mononuclear cells (PBMCs) derived from 89 advanced NSCLC patients were isolated before the beginning of immunotharepy  by the Ficoll-Hypaque gradient (Lympholite-H, Ontario, Canada). Concurrently, sera derived from 85 patients were purified using the BD Vacutainer Plus Plastic Serum tubes (Becton Dickinson, Franklin Lakes, New Jersey, US) by centrifugation at 1.800 rpm for 10 min. PBMCs and patients’ sera were cryopreserved until use.

### Immunophenotyping

PBMCs were immunophenotypically characterized by cytofluorimetry using a multi-parametric analysis. The following monoclonal antibodies (mAb) were used: anti-CD3-BV510 (HIT3a clone), CD8-APC-H7 (SK1 clone), CD137 (4-1BB)-APC (4B4-1 clone), PD1-BB700 (EH12.1 clone), CD45RA-BB515 (HI100 clone), Ki67-BV420 (B56 clone) (all from BD Biosciences, San Jose, CA), and CCR7-PE (G043H7 clone) (BioLegend, San Diego, CA) to analyse the different T cell subsets; anti-CD3 BV510 (HIT3A clone), anti-CD4-APC-H7 (RPA-T4 clone), CD45RA-BB515 (HI100 clone) (all from BD Biosciences), CD25-PE (MA251clone) (BioLegend), FOXP3-APC (PCH101 clone) (Thermo Fisher Scientific, Waltham, MA, USA) to study Tregs; CD66b-PeCy7 (DREG-5 clone, BD Biosciences), HLA-DR-FITCH (L243 clone), CD45-AF700 (2D1 clone), CD11c-BV421 (3.9 clone), LOX1-PE (15C4 clone), CD14-BB700 (MφP9 clone), CD15-APC (HI98 clone) (all from Biolegend) for the characterization of MDSCs. The Fluorescence minus one (FMO) and autofluorescence were used as negative controls. Zombie Aqua Fixable Viability Kit (BioLegend) was used to identify live cells and Foxp3/Transcription Factor Staining Buffer Set (Invitrogen, Waltham, MA,) was used for intracellular staining. The samples were acquired by DxFLEX Flow Cytometer (Beckman Coulter, Brea, CA) and analyzed by FlowJo (version 10.8.8, Becton Dickinson), analysis software.

### Measure of soluble immune checkpoints and cytokines

Soluble immune checkpoints and cytokines were measured using the Immuno-Oncology Checkpoint 14-Plex Human ProcartaPlex Panel and the Inflammation 20-Plex Human ProcartaPlex Panel (both from ThermoFisher Scientific) according to the manufacturer’s instructions. The 14 immune checkpoints analyzed were: BTLA, GITR, HVEM, IDO, LAG-3, PD-1, PD-L1, PD-L2, TIM-3, CD28, CD80, CD137, CD27, CD152, while the following 20 cytokines were measured: sE-Selectin, GM-CSF, ICAM/CD54, IFNα, IFNγ, IL1α, IL1β, IL4, IL6, IL8, IL10, IL12p70, IL13, IL17A/CTLA8, IP10/CXCL10, MCP1/CCL2, MIP1α/CCL3, MIP1β/CCL4, sP-Selectin, TNFα. All these factors were evaluated by Luminex multiple assays and analyzed using Bioplex Manager MP software (Bio-Rad, Hercules, CA, USA).

### Statistical analysis

A Student’s t-test was used to compare groups of continuous variables. The Kaplan and Meier method was used to calculate PFS and OS. Differences between the Kaplan and Meier curves of PFS and OS were assessed using the log-rank test. The multivariate Cox proportional hazard regression model was implemented to assess the patients’ PFS or OS predictors.

The sample size of this exploratory retrospective study will allow estimating the accuracy of Kaplan–Meier curves for PFS with a precision of approximately 15 and 20% for the whole NSCLC patient group and for naive patients treated with ICIs ± chemotherapy as first-line at 12 months in each subgroup. The significant level was defined as p-value ≤ 0.05.

Data were collected in an anonymous database and analyzed with R-package or Graphpad Prism version 7 (Graphpad Software, Inc., San Diego, CA, USA).

## Results

### Patients’ characteristics

Eighty-nine patients with metastatic NSCLC, enrolled in this study according to inclusion criteria, were described in Table 1. Fifty-five patients were treated with ICIs alone or in association with chemotherapy as first-line therapy. Twenty-four patients were treated with Pembrolizumab as monotherapy (TPS ≥ 50%), while thirty-one patients underwent immunotherapy plus chemotherapy (TPS < 50%). Thirty-four patients were treated with Nivolumab as second-line treatment regardless of TPS status. Forty-three patients had PS = 0 (48%), and 46 were classified as PS ≥ 1 (PS = 1: 36; PS = 2: 10;). Most patients were current or former smokers (88%), and 12% of patients declared never smoked. The disease control rate was used to classify responder (R) (61%) and non-responder (NR) (39%) patients. Forty-three patients had a disease progression, while 28 showed a partial response and 18 had stable disease as the best response rate.

**Table 1:** Patients’ characteristics

Tot	n° (%) Tot 89 (100)
Sex	
Male	61 (68.5)
Female	28 (31.5)
Age	
Median range	66.5
< 75	73 (82)
> 75	16 (18)
Histotype	
Adenocarcinoma	61 (68.5)
Squamous	28 (31.5)
EOCG performance status	
0	43 (48)
> 1	46 (52)
TPS	
> 50%	24 (27)
< 50%	31 (35)
Unknown	34 (38)
Therapy for metastatic disease	
* I line therapy*	
Pembrolizumab	24 (27)
Pembrolizumab + CHT	15 (17)
Ipi/Nivo + CHT	16 (18)
* II line*	
Nivolumab	34 (38)
Smoking status	
Smoker (current/former)	11 (12)
Non-smoker	78 (88)
Response to ICIs	
Yes	54 (61)
No	35 (39)
Best response rate	
Partial response	28 (31.5)
Stable disease	18 (20.5)
Progression	43 (48)

*TPS* Tumor proportion score, *CHT* chemotherapy, *Ipi* Ipilimumab, *Nivo* Nivolumab, *ICIs* Immune checkpoint inhibitors

### High levels of CD137^+^ T cell subsets were associated with response to anti-PD1 treatment

**Fig. 1:**
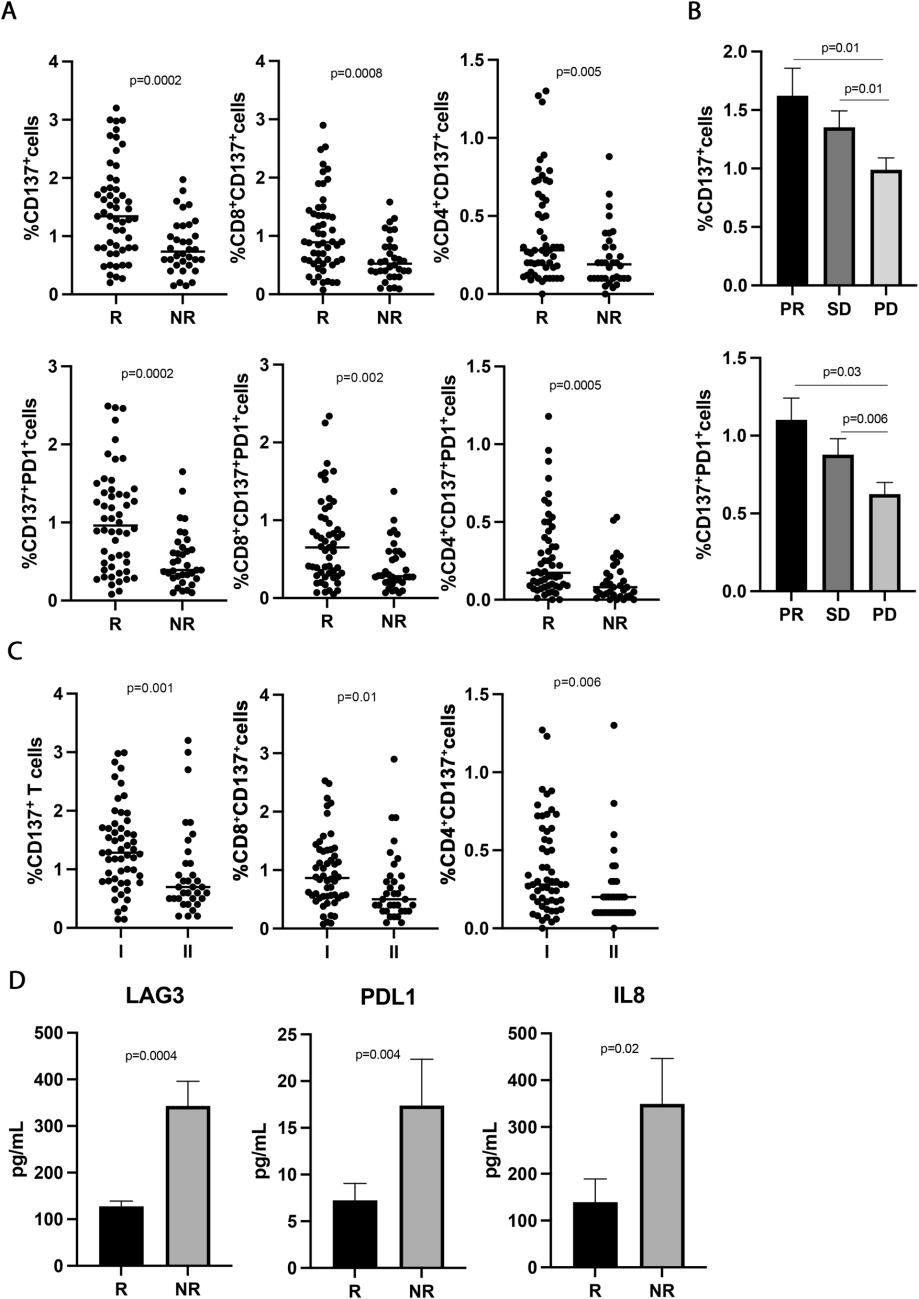
CD137^+^ T cell subsets sLAG3, sPDL1, and IL-8 correlated with response to ICIs in 89 NSCLC patients. **A** The scattered dot plots display the values of CD137^+^ (total, CD8^+^, and CD4^+^) and CD137^+^ PD1^+^ (total, CD8^+^, and CD4^+^) T cells in responder (R) and non-responder (NR) patients. The horizontal lines correspond to the median values of each CD137^+^ T cell subset in the two groups of patients. **B** The histograms represent the median values of CD137^+^ and CD137^+^ PD1^+^ T cells of patients with partial response (PR, in black), stable disease (SD, in dark grey), and progression (PD, in light grey) ± standard error of the mean (SEM). **C** The scattered dot plots show the percentage of CD137^+^ T cells (total, CD8^+^, and CD4^+^) in patients treated with immunotherapy as first (I) and second line (II). The horizontal lines correspond to the median values of each CD137^+^ T cell subset in the two groups of patients. **D** The histograms represent the median values expressed in pg/mL of soluble LAG3, PDL1, and IL8 in R (black histograms) and NR (gray histograms) patients ± SEM. *p* values < 0.05 were considered significant

To study the immune activatory profile of NSCLC patients enrolled in this study, the circulating CD137^+^ T cell subsets were analyzed. The results showed that high levels of CD137^+^ and CD137^+^PD1^+^ T cells positively correlated with response to immunotherapy (Fig. 1A). Indeed, responding patients had higher levels of CD137^+^ T cells and CD137^+^PD1^+^ T cell subsets compared to non-responders (CD137^+^, R vs. NR 1.4 ± 0.8 vs. 0.82 ± 0.4, p = 0.0002; CD137^+^PD1^+^, R vs. NR 1.03 ± 0.65 vs. 0.52 ± 36 and p = 0.0002), although the percentage of CD3^+^ and PD1^+^ T cells was similar between the two groups (Additional file 1: Figure S1A). The differences observed in the CD137^+^ and CD137^+^PD1^+^ subsets could be ascribed to both CD8 and CD4 T cell populations (CD8^+^CD137^+^, R vs. NR 1.04 ± 0.67 vs. 0.59 ± 0.37, p = 0.0008; CD4^+^CD137^+^, R vs. NR 0.39 ± 0.3 vs. 0.22 ± 0.19, p = 0.005; CD8^+^CD137^+^PD1^+^, R vs. NR 0.73 ± 0.55 vs. 0.4 ± 0.3, p = 0.002; CD4^+^CD137^+^PD1^+^, R vs. NR 0.27 ± 0.2 vs. 0.12 ± 0.1, p = 0.0005) suggesting that each of this cell subset contribute to the overall anti-tumor immune response in this setting of patients. These immunological features were also correlated to the best overall response rate (Fig. 1B). Interestingly, patients with progression (PD) displayed significantly lower levels of CD137^+^ and CD137^+^PD1^+^ T cells as compared to the groups of patients with partial response (PR) and stable disease (SD) (PR vs. PD 1.6 ± 1.2 vs. 0.9 ± 0.6, p = 0.01; SD vs. PD 1.3 ± 0.5 vs. 0.9 ± 0.6, p = 0.01) (PR vs. PD 1.1 ± 0.7 vs. 0.6 ± 0.4, p = 0.03; SD vs. PD 0.87 ± 0.4 vs.0.6 ± 0.4, p = 0.006). No difference in the percentage of these two cellular subsets was observed between patients with PR and SD. These results indicate that CD137^+^T cells as well as CD137^+^PD1^+^ T cells are strongly associated with the clinical response.

CD137^+^ T cells were also evaluated stratifying the patients according to sex (female vs. male), histotype (adenocarcinoma vs. squamous), and smoking status (smoker vs. non-smoker), however, no significant differences were observed among groups. Other T cell subpopulations (Ki67^+^ and effectors, naïve, central memory, and effector memory cells in the CD3^+^, CD137^+^, and PD1^+^ T cells) were also analyzed without statistical significance (data not shown).

Finally, the levels of CD137^+^ T cells (total, CD8, and CD4) were also assessed according to the administration of immunotherapy (first- vs. second- line) (Fig. 1C). Results showed that naïve patients had high levels of CD137^+^ T cells (total, CD8, and CD4) compared to platinum-doublet chemotherapy-treated patients, demonstrating that chemotherapy negatively impacts the immune activatory profile of cancer patients.

### High circulating levels of soluble LAG3, PDL1, and IL8 inversely correlated with response to immunotherapy

To evaluate the impact of soluble immune molecules on the response to immunotherapy, 14 checkpoints, and 20 cytokines were analyzed in the serum of NSCLC patients before therapy (Fig. 1 D). Among the soluble checkpoints evaluated, sLAG3 and sPDL-1 were inversely associated with the response to ICIs. Indeed, both molecules were higher in non-responding patients (sLAG3, R vs. NR 127.5 ± 71 vs. 343 ± 302, p = 0.0004; sPDL-1, R vs. NR 7.2 ± 12.6 vs.17.39 ± 29.3, p = 0.04), confirming their correlation with a worse clinical outcome. Concurrently, the analysis of the cytokines in the serum patients showed that only the pro-inflammatory cytokine, sIL8, correlated with response to anti-PD1 treatment and displayed a higher concentration in the non-responding group (sIL8, R vs. NR 139 ± 242 vs. 349 ± 433, p = 0.02). All these data suggested that non-responder patients had before the beginning of ICIs a worse immunological fitness than responders characterized by a higher inflammatory *milieu.* Interestingly, the release of these three molecules was correlated (sLAG3-sPDL1, r = 0.49; sLAG3-sIL8, r = 0.68; sPDL1-sIL8, r = 0.46, p < 0.001 for all correlations). These correlations were not found between these soluble mediators and the different CD137^+^ T cell subsets (Additional file 1: Figure S1C).

### CD137^+^T cell subsets correlated with longer survival

**Fig. 2:**
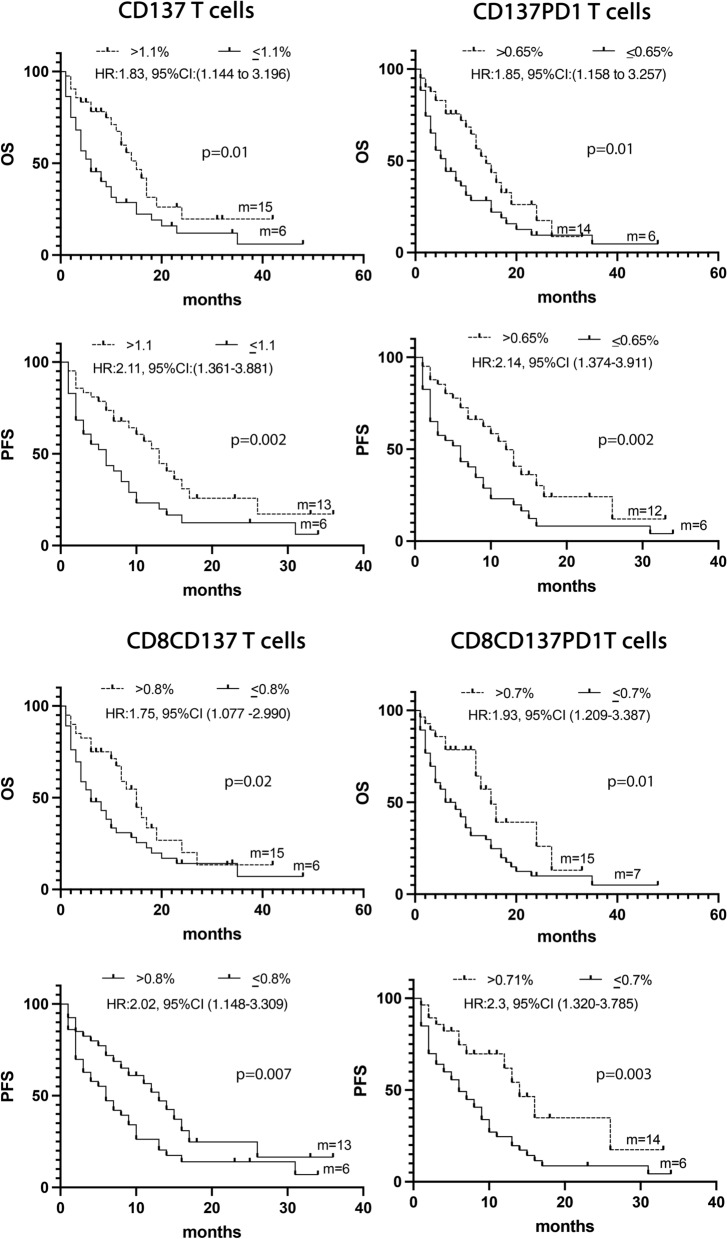
**A** Kaplan -Meier curves for PFS and OS were used to determine 1.1%, 0.65% 0.8%, and 0,7 as the cut-off of CD137^+^, CD137^+^ PD1^+^, CD137^+^CD8^+^, and CD8^+^CD137^+^ PD1^+^ T cells, respectively. Long-rack test was used to analyze the differences between the two groups. m = months, *p* values < 0.05 were considered significant

The levels of the different CD137^+^ T cell subsets together with the three soluble immune mediators and several clinical parameters such as TPS, age, sex, PS, and smoking status were analyzed by univariate analysis (UVA) to predict survival. The identified cut-off values for each biological parameter were reported in Additional file 1: Tables S1 and S2. Results depicted in Fig. 2 demonstrated that patients who showed a longer survival in terms of OS and PFS were characterized by high levels of CD137^+^ (> 1.1%), CD137^+^PD1^+^ (> 0.65%), CD8^+^CD137^+^ (> 0.8%), or CD8^+^CD137^+^PD1^+^ (> 0.7%) T cells. Moreover, the univariate analysis of clinical parameters demonstrated that both the female sex and PS = 0 correlated with a prolonged PFS (female vs. male: HR:1.78, 95% CI (1.0536–3.1015), p = 0.03; PS = 0 vs. PS > 0: HR:0.39, 95% CI (0.1818–0.5518), p < 0.0001) and OS (female vs. male: HR:1.87, 95% CI (1.1068–3.2408), p = 0.02; PS = 0 vs. PS > 0: HR:0.4, 95% CI (0.1972–0.5859), p < 0.0001) (Additional file 1: Tables S1 and S2).

Multivariate analysis confirmed that the immunological markers CD137^+^ and CD137^+^PD1^+^ T cells and the clinical parameters PS = 0, and female sex represented four positive prognostic factors of OS (p = 0.0001). All these parameters, except for CD137^+^ T cells, resulted also as positive prognostic factors of PFS (Additional file 1: Tables S3 and S4).

### CD137^+^ T cell subsets were associated with response to therapy and longer survival in patients treated with ICIs as first-line treatment

**Fig. 3:**
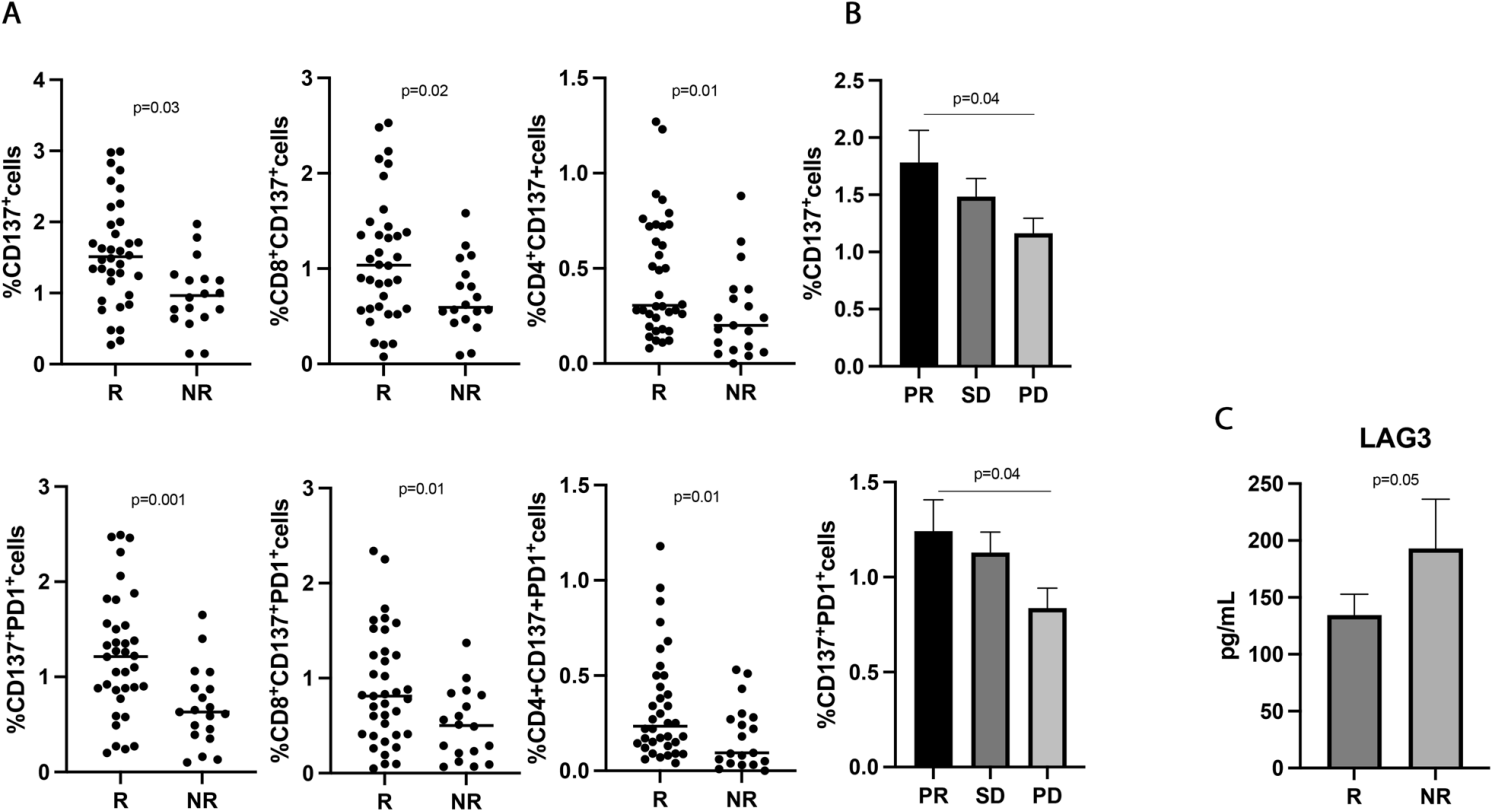
CD137^+^ T cell subsets and sLAG3, were associated with response to ICIs in 55 NSCLC patients treated with immunotherapy as upfront treatment. **A** The scattered dot plots display the values of CD137^+^ (total, CD8^+^, and CD4^+^) and CD137^+^ PD1^+^ T cells in responder (R) and non-responder (NR) patients. **B** The histograms represent the median values of CD137^+^ and CD137^+^ PD1^+^ in patients with partial response (PR, in black), stable disease (SD, in dark grey), and progression (PD, in light grey) ± standard error of the mean (SEM). **C** The histograms showed the median values expressed in pg/mL of soluble LAG3 in R (black histograms) and NR (gray histograms) patients ± SEM. *p* values < 0.05 were considered significant

The predictive and prognostic role of the different CD137^+^ T cell subsets were further analyzed in the 55 NSCLC patients treated with immunotherapy ± chemotherapy as first-line treatment (Fig. 3). This analysis is particularly important because PD1 axis blockade as monotherapy or in combination represents the standard of care for metastatic non-oncogene addicted NSCLC patients [6–8].

Results partially confirmed the data obtained in the entire population of NSCLC patients. Indeed, the CD137^+^ and CD137^+^PD1^+^ T cells, both CD8 and CD4, were associated with response to ICIs (Fig. 3A) (CD137^+^ T, R vs. NR 1.56 ± 0.7 vs. 0.97 ± 0.4, p = 0.03; CD137^+^PD1^+^ T, R vs. NR 1.22 ± 0.63 vs. 0.68 ± 41 and p = 0.001), and patients with a partial response had higher levels of both T cell subsets compared to progressors (Fig. 3B) (CD137^+^ T: PR vs. PD 1.78 ± 1.2 vs. 1.1 ± 0.6, p = 0.04; CD137^+^PD1^+^ T: PR vs. PD 1.2 ± 0.7 vs. 0.8 ± 0.5, p = 0.04). No differences were observed between patients with SD and PD and between PR and SD. Among the soluble factors, only the immune suppressive molecule sLAG3 appeared to be higher in non-responder patients, even if this difference is not statistically significant (R vs. NR 112 ± 49.5 vs. 137 ± 44, p = 0.05) (Fig. 3C).

**Fig. 4:**
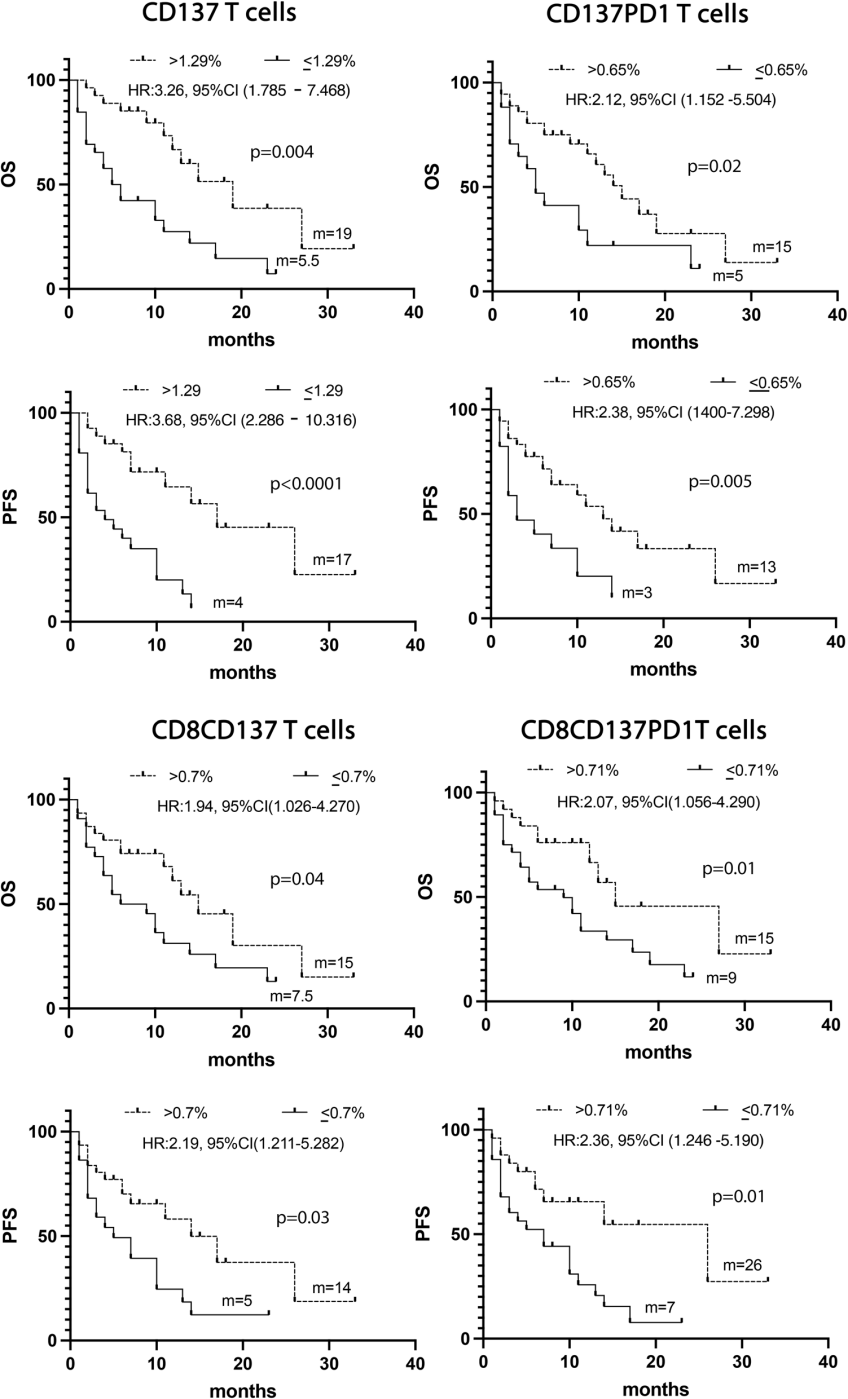
Kaplan -Meier curves for PFS and OS were used to determine 1.29%, 0.65%, 0.7%, 0.71% as the cut-off of CD137^+^, CD137^+^ PD1^+^, CD137^+^CD8^+^, and CD8^+^CD137^+^ PD1^+^ T cells. Long-rack test was used to analyze the differences between the two groups m = months, *p* values < 0.05 were considered significant

The UVA analysis, carried out considering the biological and clinical parameters, confirmed that the levels of CD137^+^ (> 1.29%), CD137^+^ PD1^+^(> 0.65%), CD8^+^CD137^+^ (> 0.71%) and CD8^+^CD137^+^PD1^+^ (> 0.71%) T cells together with PS = 0 and female sex were associated with a prolonged OS and PFS (Fig. 4 and Additional file 1: Tables S5 and S6).

Combining all these significant data in a multivariate analysis confirms the CD137^+^T cell subset as a positive prognostic factor of survival (Additional file 1: Tables S7 and S8).

### Low levels of immune suppressive PMN(Lox1^+^)-MDSCs were associated with response to immunotherapy in naïve NSCLC patients

**Fig. 5:**
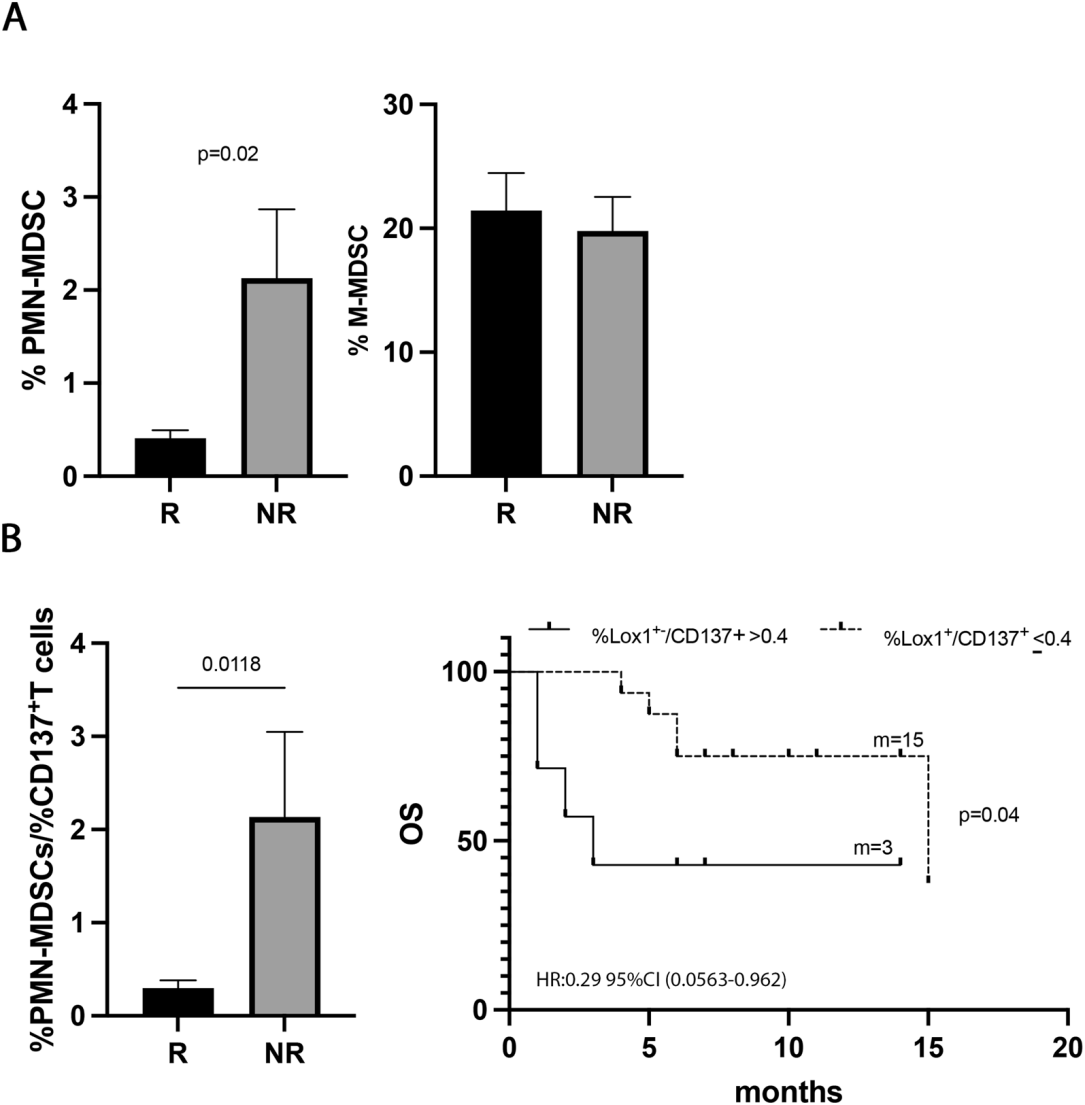
PMN (Lox1^+^)-MDSCs correlated with response to ICIs in naïve patients. **A** Histograms showed the percentage of PMN(Lox1^+^)-MDSCs and M-MDSCs in 30 naïve NSCLC patients. Black histograms represented the values of these cells in responders (R) and gray histograms represented the PMN (Lox1^+^)-MDSCs and M-MDSCs values in non-responders (NR). **B** Histogram displayed the % PMN(Lox1^+^)-MDSCs/%CD137^+^ T cell ratio in R (black) and NR (grey) naïve patients; Kaplan–Meier curves for OS were used to determine 0.4% as the cut-off of % PMN(Lox1^+^)-MDSCs/%CD137^+^T cell ratio. m = months, *p* values < 0.05 were considered significant

To understand the impact of the immunosuppressive cells in the response to ICIs, the percentage of MDSCs, both PMN(Lox1^+^)- and M-MDSCs, was evaluated in 30 NSCLC patients treated with ICIs as first-line treatment (Fig. 5). Results showed that the PMN(Lox1^+^)-MDSCs were higher in non-responder patients (R vs. NR 0.33 ± 0.3 vs. 2.1 ± 2, p = 0.02) (Fig. 5A), while the M-MDSCs seemed not to impact the response to therapy. However, no association with PFS or OS was found for the two MDSC subsets (data not shown), thus apparently suggesting that ICI treatment did not affect these two cellular subsets regarding survival.

Interestingly, the PMN(Lox1^+^)-MDSCs appeared to be associated with the clinical response when we evaluated the balance between this immune suppressor myeloid subset and the activatory CD137^+^T cells (Fig. 5B). Indeed, a low %PMN(Lox1^+^)-MDSCs/%CD137^+^T cells ratio identified responder patients (R vs. NR 0.29 ± 0.3 vs. 2.1 ± 2.2, p = 0.01) and patients with a longer OS (ratio ≤ 0.4; p = 0.04). All these data suggested that the levels of immunosuppressive PMN(Lox1^+^)-MDSCs alone were not critical for survival, however, the balance between PMN(Lox1^+^)-MDSCs and CD137^+^ T cells is relevant in determining the clinical outcome.

### The levels of circulating Tregs affected the survival of naïve NSCLC patients

**Fig. 6:**
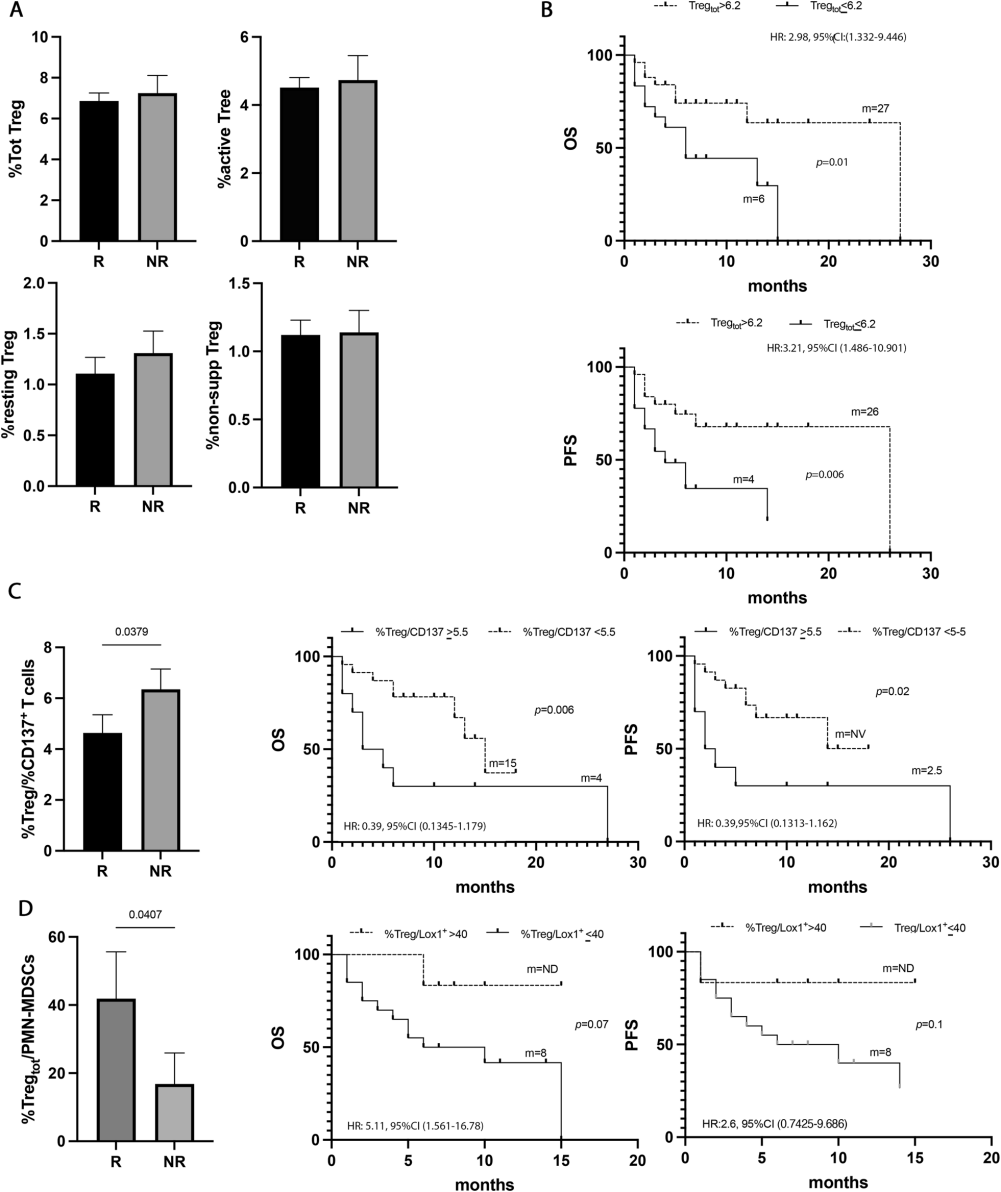
Tregs did not correlate with response to ICIs, but with survival. **A** Histograms represent the percentage of total, active, resting, and non-suppressive Tregs in NSCLC patients under immunotherapy treatment as the first line. Black histograms showed the median levels of the different populations of Tregs in responder patients (R), and the gray histograms represented the median values of non-responders (NR). **B** Kaplan Meier curves of PFS and OS of total Tregs evaluated at baseline using as cut-off a value of 6.2%. Long-rank test was used to analyze the differences between the two groups. **C** Histogram represented the ratio of %total Treg/%CD137^+^ Tcells in responders (black histogram) and non-responders (gray histogram); Kaplan Meier curves of OS and PFS of %total Treg/%CD137^+^Tcells ratio evaluated at baseline using as cut-off a value of 5.5%. Long-rank test was used to analyze the differences between the two groups. **D** Histogram represented the ratio of %total Treg/%CD137^+^ T cells in responders (black histogram) and non-responders (gray histogram); Kaplan Meier curves of OS and PFS of %total Treg/%PMN(Lox1^+^)-MDSC ratio evaluated at baseline using as cut-off a value of 40%. Long-rank test was used to analyze the differences between the two groups. m = months. *p* values < 0.05 were considered significant

To analyze the role of Treg cells in NSCLC, the percentage of Tregs, (total, non-suppressive, resting, and active) was evaluated in all the naïve patients (Fig. 6). Tregs and their subpopulations appeared not to affect the response to immunotherapy (Fig. 6A). However, when these populations were analyzed regarding survival, a high percentage of total Tregs (> 6.2%) appeared to be correlated with longer survival in terms of OS and PFS (p = 0.02 and p = 0.01, respectively) (Fig. 6B, Additional file 1: Tables S5 and S6). Combining these significant results in the multivariate analysis emerged that Tregs is another favorable prognostic factor of survival in this setting of patients (Additional file 1: Tables S7 and S8).

Moreover, analyzing the %Tregs/%CD137^+^ T cells ratio (Fig. 6C), non-responders showed a more pronounced state of immune suppression compared to responders (NR vs. R vs. 6.3 ± 2.5 vs. 4.6 ± 3.4 p = 0.04). These data were confirmed by the analysis of survival in which patients with a %Tregs/%CD137^+^ T cells ratio > 5.5% showed poor OS (p = 0.006) and PFS (0.02).

The Treg levels were also analyzed as a ratio in combination with the PMN(LOX1^+^)-MDSCs levels. Results showed that responder patients had a higher ratio compared to non-responders (R vs. NR 41.8 ± 58 vs. 16.8 ± 25.7, p = 0.04) suggesting that the levels of Tregs in the responding group prevailed among the immunosuppressive cells (Fig. 6D). Survival analysis displayed a longer OS in patients with a %Treg/%PMN(Lox1^+^)-MDSCs ratio > 40, even if this difference is not statistically significant (p = 0.07). No significant differences were observed for PFS.

## Discussion

The immune fitness of cancer patients is crucial for the success of immunotherapeutic treatments. Patients with a compromised immune system poorly respond to immunotherapy, on the contrary, patients with high levels of immune activation seem more prone to benefit from immunological therapies, showing longer survival [14]. The identification of novel immune biomarkers among the different components of the immune system able to well define the immunological status of patients, is becoming of increasing interest for the management of cancer patients.

In this study, we concurrently analyzed, before the beginning of immunotherapy, the immune fitness of 89 advanced NSCLC patients treated with ICIs and the internal cohort of 55 naïve patients who underwent immunotherapy as first-line treatment to identify new biomarkers to improve clinical intervention. This latter analysis is particularly relevant because this schedule of treatment represents today the standard of care for advanced non-oncogene addicted NSCLC patients.

Among the activated immune cells, we proposed CD137^+^ and CD137^+^PD1^+^ T cell subsets (total, CD8^+^ and CD4^+^) as biomarkers of response to ICIs in both groups of patients analyzed, suggesting that these immune subsets are critical parameters for the success of ICIs independently by the administration of previous therapies. Moreover, their levels were relevant also for patient survival, since all these populations correlated with a longer survival except for CD4^+^ T cells, confirming the primary role of CD137^+^ T cells, particularly the CD8^+^ subset in the induction of the anti-tumor immune response. Interestingly, the levels of CD137^+^ T cells decreased after chemotherapy. Despite this decrease, the levels of CD137^+^ T cells remained higher in both groups of responding patients (I and II line) confirming its role as a predictive biomarker in these different settings of patients (Additional file 1: Figure S2).

We finally identify the entire CD137^+^ T cell population, and in particular the most functionally active CD137^+^PD1^+^ T cell subset as two favorable prognostic factors of survival in the entire population of NSCLC patients. By combining this cellular parameter with sex and performance status, it was possible to identify those patients with longer survival, suggesting that a better clinical status correlated with the wellness of the immune system. The prognostic significance of CD137^+^ T cells was also validated in the group of naïve patients, confirming that high levels of activated immune cells before the beginning of immunotherapy could represent an appealing predictive biomarker.

In line with our results, several other studies confirmed the role of circulating CD137^+^ lymphocytes as biomarkers. In melanoma patients, cytotoxic CD137^+^ T cells correlated with a disease-free status [22]. In metastatic renal carcinoma, CD137^+^ T cells were associated with the clinical response to tyrosine kinase inhibitors [23, 24]. We recently demonstrated that the amount of circulating CD137^+^ T cells had a predictive and prognostic role in survival in advanced head and neck squamous cell carcinoma (HNSCC) patients treated with pembrolizumab as first-line therapy [15]. In another study carried out on 109 patients with different solid tumors under anti-PD1 treatment, we demonstrated and validated that high levels of CD137^+^ T cells were a prognostic factor of PFS and OS. In the same study, we showed that a high percentage of the more activated CD137^+^PD1^+^ T cells was associated with the response to therapy and survival and that the CD137^+^CD8^+^PD1^+^ T cells represented the more proliferative subset compared to the CD137^−^CD8^+^PD1^+^ ones [14]. All these data highlight the important implication of CD137^+^ T cell populations in the anti-tumor immune response and confirm the role of these populations as optimal predictive and prognostic biomarkers not only in the setting of NSCLC.

All NSCLC patients and the internal cohort of naïve patients were also examined for the presence of soluble cytokines and immune checkpoints. Among the immune checkpoints, sLAG3 appeared the only parameter able to affect the response to therapy in both populations. Indeed, we demonstrated that sLAG3 was higher in non-responder patients, suggesting that this molecule contributes to the impairment of the immunotherapy, independently by previous therapy. Our analysis was confirmed by the results obtained in other solid tumors such as HNSCC and melanoma, in which sLAG3 appeared to identify patients with a worse prognosis or resistance to treatment [25, 26]**.** Moreover, in addition to sLAG3, sPDL1 and sIL8 resulted as predictive biomarkers of response only when the analyses were carried out in the overall NSCLC population, suggesting that the release of these soluble molecules could be induced by other therapies such as chemotherapy. Both these factors were higher in non-responder patients conferring to both molecules a negative role in the regulation of the immune response. sIL8 is largely investigated in NSCLC. Several authors suggested that chemotherapy caused inflammatory changes inducing the release of a large amount of sIL8 that could confer drug resistance [27, 28]. Moreover, other authors demonstrated that serum levels of sIL8 could be used to predict the response to ICIs [29]. Concerning the sPDL1, its release was correlated with a poor prognosis [14] and seemed to be tumor-type dependent. Moreover, the increase in serum levels of sPDL1 during ICIs appeared to be therapy-specific [30]. All these data suggest that despite soluble immune molecules may be a key point in regulating the patient’s immune system by contributing directly to modifying the activation state of the immune system their employment as possible biomarkers can be limited.

The response to immunotherapy strictly correlates with the balance between immune activation and suppression. Therefore, the naïve patient group was also analyzed for the levels of immunosuppression by evaluating the amount of circulating MDSCs and Tregs. These two cellular subsets play a crucial role in cancer patients promoting tumor growth and affecting the efficacy of immunotherapy [31, 32]. Here, we found that the levels of PMN(Lox1^+^)-MDSCs, which represent the most immunosuppressive MDSC subset, inversely correlated with clinical response. Moreover, non-responding patients showed an imbalance in the immune system towards immune suppression displaying a high %PMN(Lox1^+^)-MDSCs/%CD137 ^+^ T cell ratio that suggests the presence of a worse immunological status in non-responding patients. The negative effect on the clinical outcome of high circulating levels of PMN(Lox1^+^)-MDSCs was confirmed by other authors in different solid tumors [33, 34]. In the NSCLC setting, the amount of circulating and infiltrating ^+^PMN(Lox1^+^)-MDSCs seemed to be linked to a poor prognosis [35]. Their recruitment in tumor sites was higher in non-responder patients and further increased after anti-PD1 therapy suggesting that PMN(Lox1^+^)-MDSCs could be a specific subset with immunosuppressive function in this setting of patients [13]. The pivotal role of PMN(Lox1^+^)-MDSCs in determining the immunosuppression halting the efficacy of ICI treatment was also confirmed by the balance with Tregs. Indeed, non-responder patients with reduced OS were characterized by a high proportion of PMN(Lox1^+^)-MDSCs compared to Tregs (%PMN(Lox1^+^)-MDSCs/%Tregs > 40).

This evidence was further confirmed by the analysis of Tregs in which a high percentage of total Tregs was correlated with longer OS and resulted as a favorable prognostic factor. These results are in agreement with recent studies reporting that melanoma and NSCLC patients responding to ICIs showed higher levels of circulating Tregs than non-responders and that a high amount of Tregs at baseline was associated with a favorable prognosis to anti-PD1 therapy [13, 36, 37].

The diverse distribution of Tregs in the peripheral blood and the tumor bed appears to be critical for these cells to exert their immunosuppression function. In fact, in an NSCLC tumor model, the number of circulating Tregs was inversely correlated with tumor-infiltrating Tregs [13], thus suggesting that is the infiltrating Tregs that plays a fundamental role in anti-tumor failure. However, the prognostic significance of circulating Tregs in solid tumors is today largely debated because different results have been obtained according to the stage of disease and the histological type of tumor. In addition, infiltrating Treg appeared to have a profile of activatory and inhibitory receptors associated with a more severe immunosuppressive capacity compared to those expressed by circulating Treg [38].

In this complex scenario, our results indicate that the combined analysis of peripheral Tregs and CD137^+^ T cells allows a more robust identification of immune balance to be employed as a biomarker of survival. In fact, despite the apparent beneficial prognostic role of a high amount of Tregs in the peripheral blood, patients with high levels of CD137^+^ T cells compared to Tregs showed a longer survival indicating that the balance between immune activation and immune suppression is fundamental for establishing the type of immune response provoked and in determining the clinical outcome of cancer patients. Therefore, we propose the %Treg/%CD137^+^ T ratio as a novel immune biomarker for predicting the survival of NSCLC patients undergoing the actual gold standard treatment.

In conclusion, this study identified CD137^+^, CD137^+^PD1^+^ T cells (total, CD4 and CD8), and the soluble immune checkpoint LAG3 as biomarkers of response to therapy in NSCLC patients under immunotherapy treatment. In the cohort of naïve patients, although the limited number of patients, we propose the CD137^+^ T cells and Tregs as two positive prognostic factors and the %Tregs/%CD137^+^ T ratio, as a novel biomarker to identify patients with longer survival. We strongly believe that these analyses could be particularly relevant in the identification of novel parameters aiding the clinicians to overcome all the critical steps derived from the evaluation of tPDL1 (location of the biopsy, timing of analysis, PDL-1 heterogeneity) and to monitor the changes of the immune system during therapy predicting the efficacy of treatment and resistance. Further studies in this direction (immune-monitoring) must be carried out to validate these biomarkers, improve immunological characterization of cancer patients during therapy, and provide oncologists with new parameters that can support the optimal choice of therapy.

### Supplementary Information

**Additional file1: Figure S1.** Analysis of T cell subsets and correlation between soluble factors and immune cell subsets. A. Scatter dot plots corresponding to CD3, CD8, CD4, PD1, CD8PD1, and CD4PD1 T cells evaluated by cytofluorimetry in 89 NSCLC patients undergone ICIs as first or second-line treatment. B. Scattered dot plots show the levels of effector (CCR7^-^CD45RA^+^), naive (CCR7^+^CD45RA^+^), central memory (CCR7^+^CD45RA^-^), effector memory (CCR7^-^CD45RA^-^), and Ki67^+^ T cells in NSCLC patients. Bars corresponding to the media values . The analysis was carried out in responding (R) and non-responding patients (NR). In all these T cell populations, no significant difference between R and NR was found. C) Correlation between immune cells and soluble factors. Each square displays the r value. Red and green squares identify the positive and negative correlation, respectively. **Figure S2.** Responders who underwent ICIs as first or second-line treatment show high levels of CD137^+^ T cells compared to non-responders. Histograms represent the median levels of CD137^+^ T cell subset in responding (R) and non-responding (NR) patients who underwent immunotherapy as first- and second-line. P<0.05 were considered statistically significant.**Table S1.** Univariate analysis of progression-free survival evaluating the entire NSCLC patients. **Table S2.** Univariate analysis of overall survival evaluating the entire NSCLC patients. **Table S3.** Multivariate analysis of PFS evaluating t the overall NSCLC population. **Table S4.** Multivariate analysis of OS evaluaing the overall NSCLCpopulation.**Table S5.** Univariate analysis of progression-free survival evaluating NSCLC patients treated with ICI treatment as first-line. **Table S6.** Univariate analysis of overall survival evaluating NSCLC patients treated with ICI treatments as first-line. **Table S7.** Multivariate analysis of PFS evaluatin the NSCLC patients treated with ICI treatments as first-line. **Table S8.** Multivariate analysis of overall survival evaluating NSCLC patients treated with ICI treatments as first-line.

## Data Availability

Data were generated by the authors and included in the article.
