# Supplementary material for: CD137+ and regulatory T cells as independent prognostic factors of survival in advanced non-oncogene addicted NSCLC patients treated with immunotherapy as first-line
Source: J Transl Med. 2024 Apr 3;22:329. doi: 10.1186/s12967-024-05142-6 (PMC10993529; doi:10.1186/s12967-024-05142-6)
Supplement: Supplementary file 1 — Additional file1: Figure S1. Analysis of T cell subsets and correlation between soluble factors and immune cell subsets. A. Scatter dot plots corresponding to CD3, CD8, CD4, PD1, CD8PD1, and CD4PD1 T cells evaluated by cytofluorimetry in 89 NSCLC patients undergone ICIs as first or second-line treatment. B. Scattered dot plots show the levels of effector (CCR7-CD45RA+), naive (CCR7+CD45RA+), central memory (CCR7+CD45RA-), effector memory (CCR7-CD45RA-), and Ki67+ T cells in NSCLC patients. Bars corresponding to the media values . The analysis was carried out in responding (R) and non-responding patients (NR). In all these T cell populations, no significant difference between R and NR was found. C) Correlation between immune cells and soluble factors. Each square displays the r value. Red and green squares identify the positive and negative correlation, respectively. Figure S2. Responders who underwent ICIs as first or second-line treatment show high levels of CD137+ T cells compared to non-responders. Histograms represent the median levels of CD137+ T cell subset in responding (R) and non-responding (NR) patients who underwent immunotherapy as first- and second-line. P<0.05 were considered statistically significant.Table S1. Univariate analysis of progression-free survival evaluating the entire NSCLC patients. Table S2. Univariate analysis of overall survival evaluating the entire NSCLC patients. Table S3. Multivariate analysis of PFS evaluating t the overall NSCLC population. Table S4. Multivariate analysis of OS evaluaing the overall NSCLCpopulation.Table S5. Univariate analysis of progression-free survival evaluating NSCLC patients treated with ICI treatment as first-line. Table S6. Univariate analysis of overall survival evaluating NSCLC patients treated with ICI treatments as first-line. Table S7. Multivariate analysis of PFS evaluatin the NSCLC patients treated with ICI treatments as first-line. Table S8. Multivariate analysis of overall survival evaluating NS [file 12967_2024_5142_MOESM1_ESM.pptx]

## Slide 1
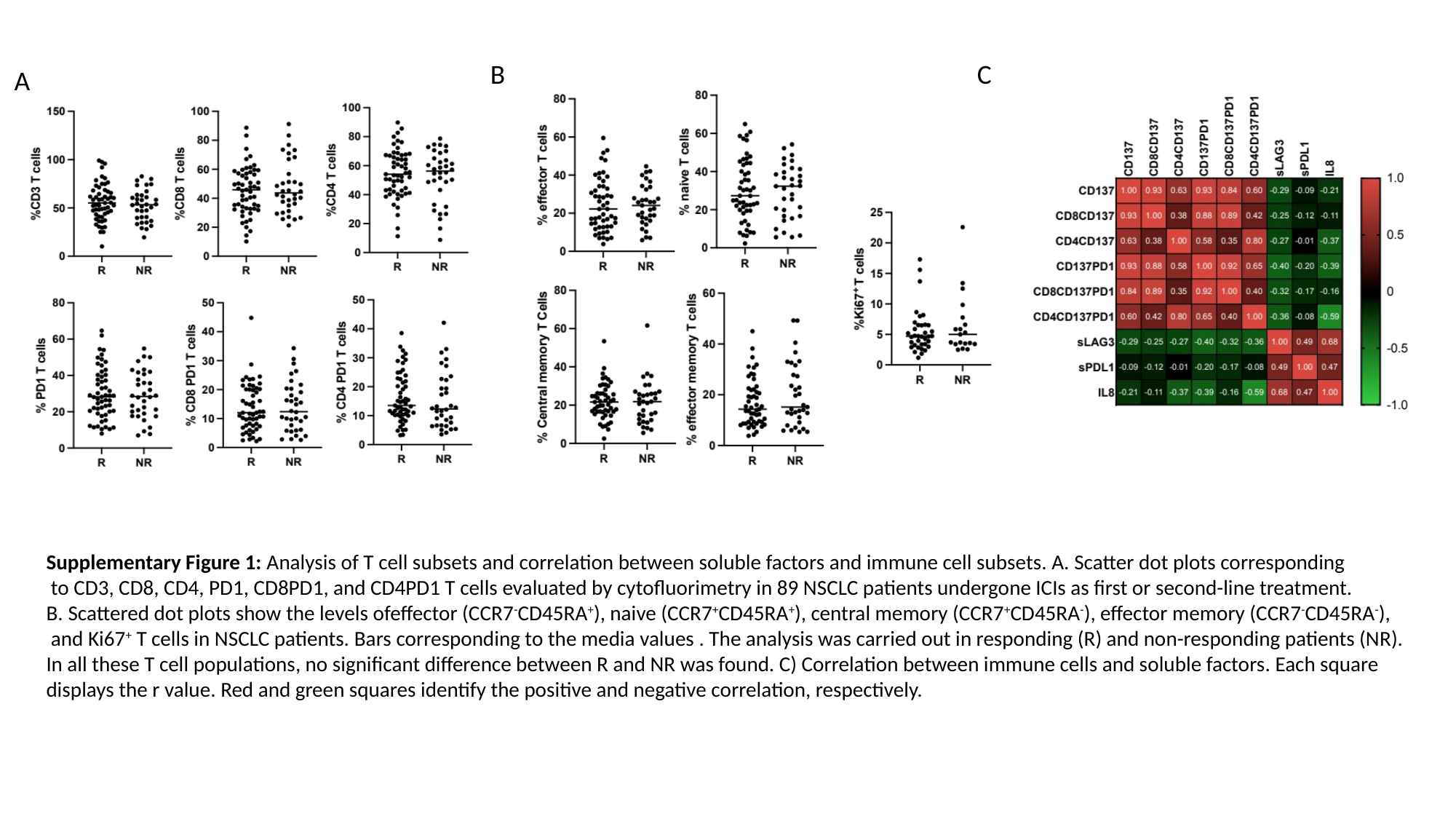

B
C
A
Supplementary Figure 1: Analysis of T cell subsets and correlation between soluble factors and immune cell subsets. A. Scatter dot plots corresponding
 to CD3, CD8, CD4, PD1, CD8PD1, and CD4PD1 T cells evaluated by cytofluorimetry in 89 NSCLC patients undergone ICIs as first or second-line treatment.
B. Scattered dot plots show the levels ofeffector (CCR7-CD45RA+), naive (CCR7+CD45RA+), central memory (CCR7+CD45RA-), effector memory (CCR7-CD45RA-),
 and Ki67+ T cells in NSCLC patients. Bars corresponding to the media values . The analysis was carried out in responding (R) and non-responding patients (NR).
In all these T cell populations, no significant difference between R and NR was found. C) Correlation between immune cells and soluble factors. Each square
displays the r value. Red and green squares identify the positive and negative correlation, respectively.

## Slide 2
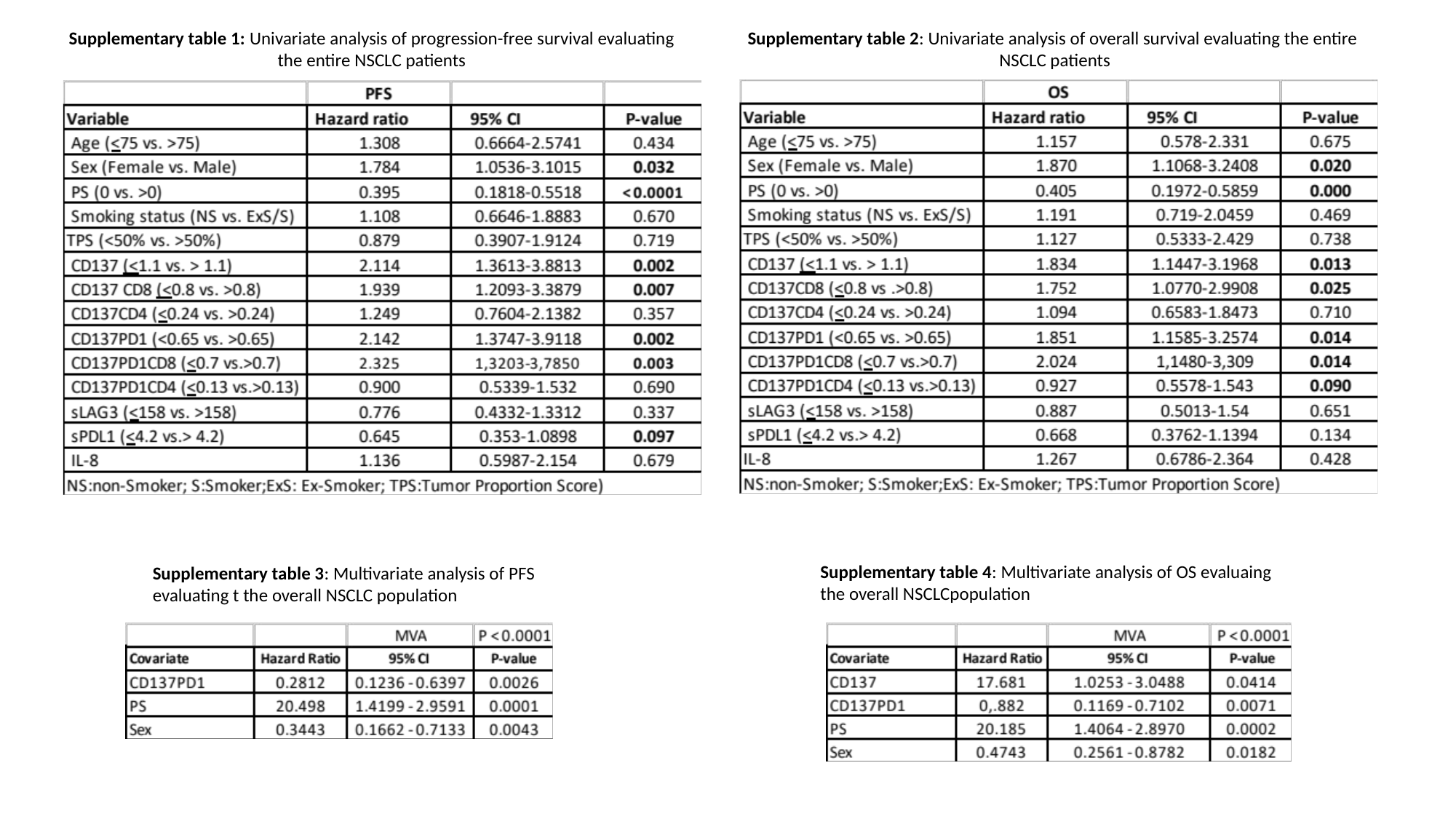

Supplementary table 1: Univariate analysis of progression-free survival evaluating the entire NSCLC patients
Supplementary table 2: Univariate analysis of overall survival evaluating the entire
NSCLC patients
Supplementary table 4: Multivariate analysis of OS evaluaing the overall NSCLCpopulation
Supplementary table 3: Multivariate analysis of PFS evaluating t the overall NSCLC population

## Slide 3
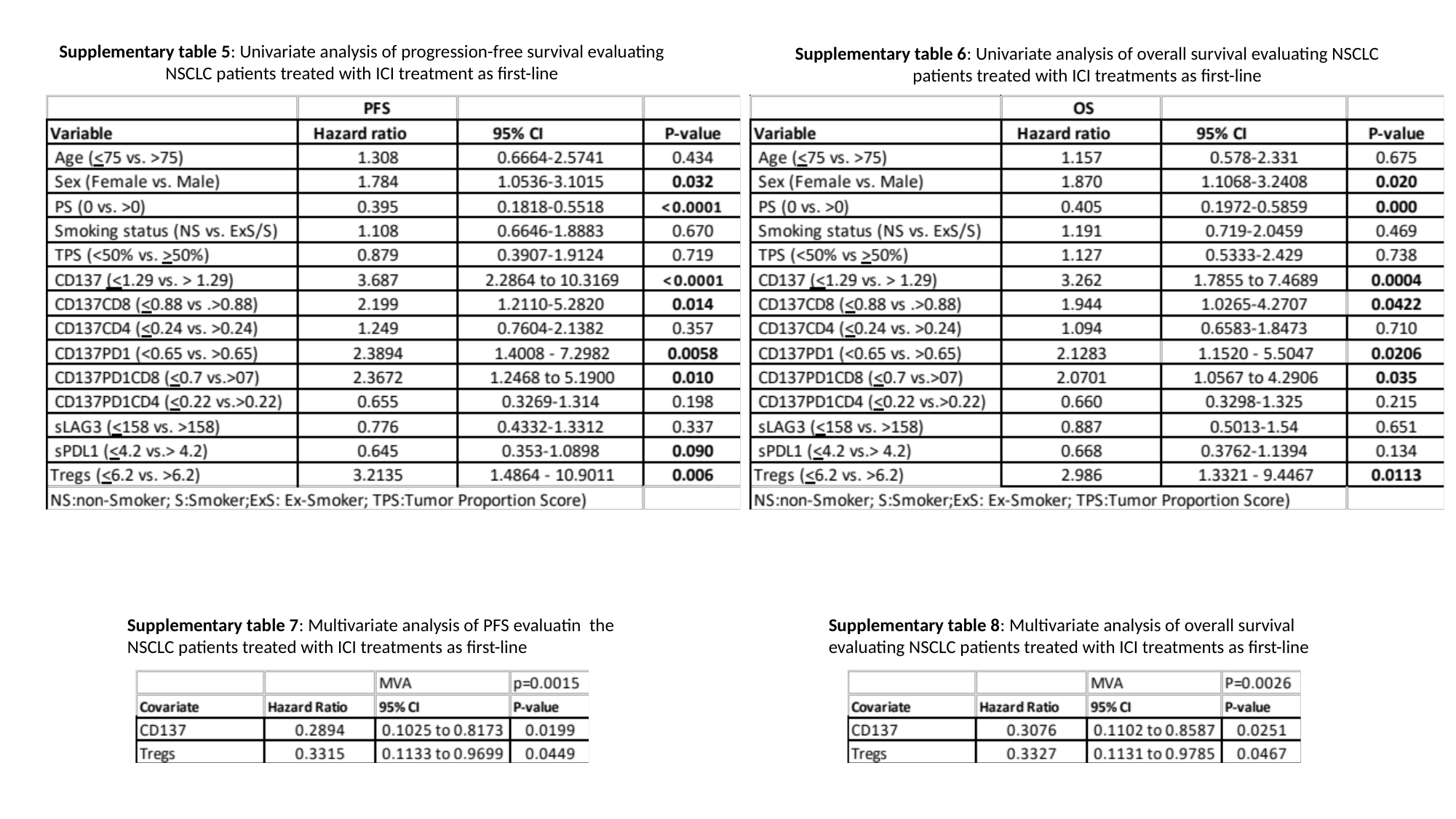

Supplementary table 5: Univariate analysis of progression-free survival evaluating NSCLC patients treated with ICI treatment as first-line
Supplementary table 6: Univariate analysis of overall survival evaluating NSCLC patients treated with ICI treatments as first-line
Supplementary table 7: Multivariate analysis of PFS evaluatin the NSCLC patients treated with ICI treatments as first-line
Supplementary table 8: Multivariate analysis of overall survival
evaluating NSCLC patients treated with ICI treatments as first-line

## Slide 4
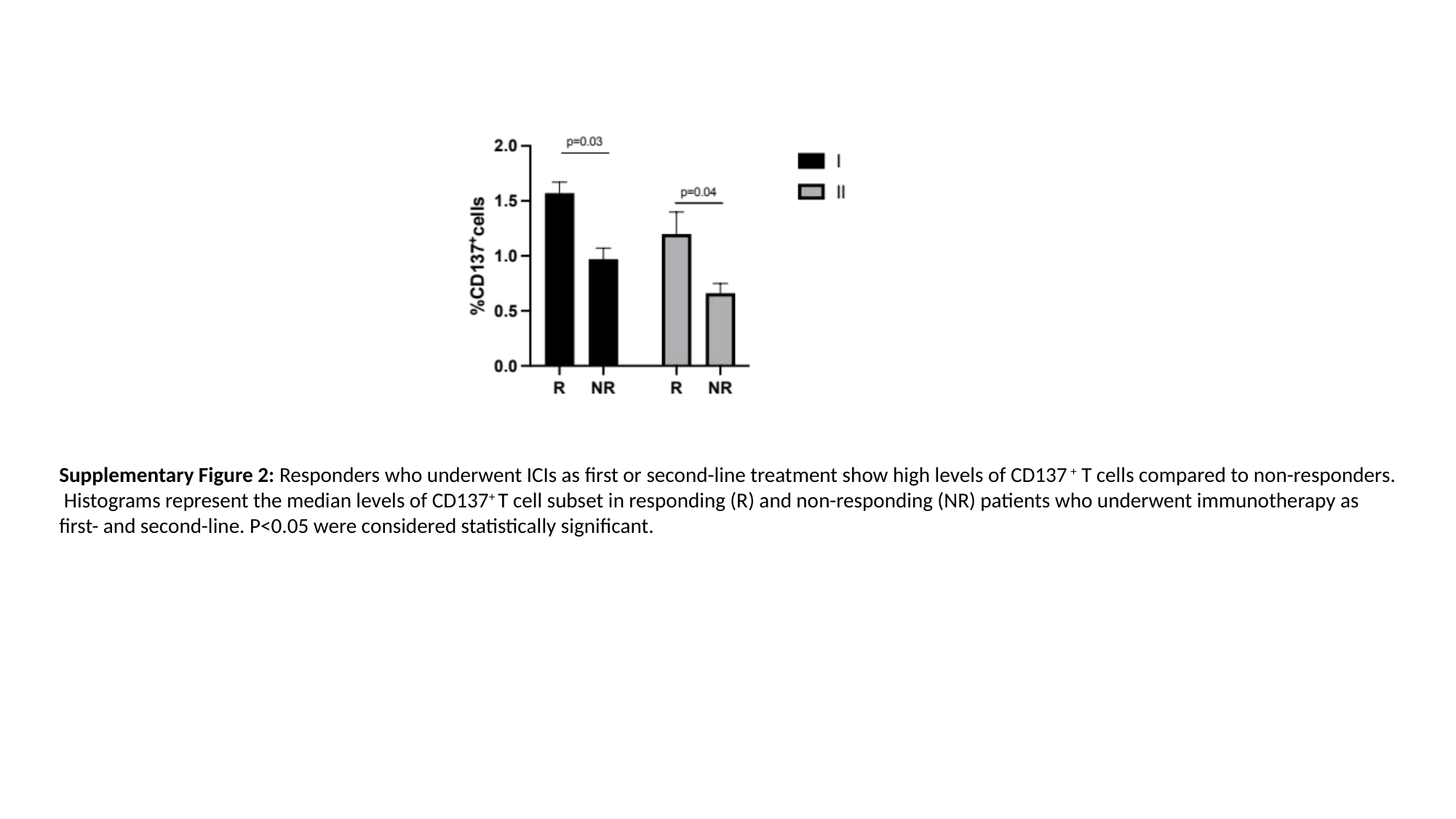

Supplementary Figure 2: Responders who underwent ICIs as first or second-line treatment show high levels of CD137 + T cells compared to non-responders.
 Histograms represent the median levels of CD137+ T cell subset in responding (R) and non-responding (NR) patients who underwent immunotherapy as
first- and second-line. P<0.05 were considered statistically significant.
